# A Pilot Study of Metacognitive Training in U.S. Republican Leaners: Reducing Polarization Toward LGBTIQ+ Persons

**DOI:** 10.1007/s10508-024-02856-y

**Published:** 2024-04-22

**Authors:** Klaus Michael Reininger, Helena Koulen, Hannah Marie Biel, Timo Hennig, Laura Pietras, Martin Rochus Kokot, Bernd Löwe, Peer Briken, Steffen Moritz

**Affiliations:** 1 Department for Psychosomatic Medicine and Psychotherapy, University-Medical Center Hamburg-Eppendorf, Martinistr. 52, 20246 Hamburg, Germany; 2 Institute of Psychotherapy, University-Medical Center Hamburg-Eppendorf, Hamburg, Germany; 3 Faculty of Human Sciences, Department of Inclusive Education, University of Potsdam, Potsdam, Germany; 4 Institute for Sex Research Sexual Medicine and Forensic Psychiatry, University-Medical Center Hamburg-Eppendorf, Hamburg, Germany; 5 Department for Psychiatry and Psychotherapy, University-Medical Center Hamburg-Eppendorf, Hamburg, Germany

**Keywords:** Metacognitive training, Homonegativity, Metacognitive doubt, Depolarization, Republicans, LGBTIQ+

## Abstract

Negative attitudes and stigmatization toward sexual minorities is a cause of minority stress of non-heterosexual persons on an individual level and has a negative impact on democratic coexistence in postmodern, plural society on a societal level. Derived from clinical research, we developed a short metacognitive training (MCT) intended to induce doubt toward inaccurate beliefs about LGBTIQ+ persons. We expected this MCT to reduce homonegativity, threat perceptions of LGBTIQ+ persons, and to foster extended outgroup tolerance compared to an education and a no-treatment control condition. We tested this hypothesis in U.S. Republican leaners who represent a social group that is likely to hold homonegative attitudes. We randomly assigned 490 U.S. Republican leaners to an MCT condition comprising 16 questions and respective answers (*n* = 166) vs. an education control condition (*n* = 164) vs. a no-treatment control condition (*n* = 160). We found that Republican leaners after receiving MCT (1) had a significant reduction of homonegativity (*d*s ≥ 0.28), (2) significantly perceived LGBTIQ+ persons as less threatening (*d*s ≥ 0.30), and (3) were significantly more tolerant of various outgroups such as LGBTIQ+ persons, feminists, liberals, and climate activists (*d*s ≥ 0.23) relative to both control conditions. The small effects of this short intervention and the possibility of systematically applying MCT in social discourse to reduce homonegativity with its potential significance for LGBTIQ+ individuals’ mental health are discussed. Furthermore, we highlight this pilot study’s significance toward intervention possibilities regarding political division and polarization in postmodern, democratic societies.

## Introduction

In early 2023, the *Economist* (2023) reported that the group of extreme conservative U.S.-Americans has tripled in protests against lesbian, gay, bisexual, and transgender (LGBT) Americans and their rights. The *Economist* further reported that anti-LGBT protests by these groups accounted for two-thirds of all protests in December 2022 and occurred in six states in 2021 and 18 states in 2022. One manifestation of the ongoing political polarization in the USA can be seen in (extremely) negative attitudes and stigmatization toward as well as protests against LGBTIQ+ persons. Social polarization (in both directions) seems to be an increasing phenomenon, transcending geographical boundaries, and extending beyond the confines of Western democracies. In the context of democratic discourse in plural societies, the escalating and increased polarization from both sides we observe is an unfavorable situation, or put differently: “[…] there is the undeniable danger of escalating polarization, in the course of which people may end up ‘thinking everything to the worst’ (Arendt, 1976, p. 477, citing Martin Luther) with detrimental consequences for all members of society” (Simon et al., 2019, p. 783). By contrast, the essence of plural societies rests on the premise of a common basic understanding among their members, including mutual respect and tolerance.

In addition to the significant difficulties of social polarization processes for democratic society, it is also important not to overlook the specific minority–majority context. When minorities are confronted with affective polarization (e.g., strong negative emotions such as anger, resentment, fear, or even hatred directed toward those who hold opposing views, a sense of “us versus them”) and cognitive polarization (e.g., becoming more entrenched in existing beliefs, even in the face of contradictory evidence, only accepting information that support one’s views while dismissing or ignoring information that challenges them) from a majority outgroup, this may cause minority stress (minority stress model, MSTM: Hatzenbuehler, 2009; Meyer, 2003, 2010). With regard to the minority group of LGBTIQ+ people, it has been observed that negative attitudes and stigmatization (homonegativism: Hudson & Ricketts, 1980; Jewell & Morrison, 2010; Morrison & Morrison, 2003, 2011; Morrison et al., 2009) has been the reason of psychological distress for LGBTIQ+ persons (Hatzenbuehler et al., 2013) and may promote significantly increased self-harm and suicidality rate among LGBTIQ+ individuals (e.g., Hottes et al., 2016; Klein & Golub, 2016; Marchi et al., 2022; O’Donnell et al., 2011). The mechanisms of (societal) homonegativity on the stress experience of LGBTIQ+ people emanate (in addition to the immediate experience of homonegativity and discrimination) from the internalization of significant aspects of homonegative beliefs (internalized homonegativity, e.g., McLaren, 2016; Williamson, 2000). The stressful experiences then compromise health outcomes (American Psychological Association, 2012; Herek, 2007; Roberts et al., 2015).

LGBTIQ+ persons are not only non-heterosexual and a disadvantaged group in a “heterosexist society” (Williamson, 2000, p. 98), from which they are met with homonegativity due to the deviation from heteronormativity (Jewell & Morrison, 2010; Jewell et al., 2011; Morrison & Morrison, 2003; Morrison et al., 2009). The social embeddedness, named in the MSTM, implies other social intergroup antagonisms in addition to the heterosexual vs. non-heterosexual antagonism paving the way for discrimination. In the U.S.-American context, one of the most central groups representing a majority societal group on the traditional-conservative, religious side is members and leaners of the Republican party.

Even though acceptance of homosexuality has increased in many countries over the last 20 years (Poushter & Kent, 2020) and homonegativity cannot be attributed exclusively to conservatism, homonegativity persists especially in religious and—often intertwined—political conservative groups (Heaven & Oxman, 1999; Morrison & Morrison, 2003). In order to reduce stigma in the societal context, interventions paving the way for intellectual humility and consequently less homonegativity and outgroup tolerance should be developed and implemented. Interventions aiming to reduce homonegativity need to fulfill two tasks: On the one hand, they might reduce discrimination, and on the other hand, they might improve mental health of LGBTIQ+ individuals (for a literature review, see Jewell et al., 2011).

### Interventions Aimed at Reducing Homonegativity

In their review, Hartman et al. (2022) describe that there are three levels to reducing bipartisan animosity the cognitive level, the relational level, or the institutional level. In the area of cognitive approaches and within this category interventions aiming at correcting misconceptions, they describe as one possibility that metacognitive interventions are effective (see also Reininger et al., 2020a). The focus of the aforementioned metacognitive intervention was on changing stereotypes by eliciting doubts about outgroup derogation (“Is the outgroup really as bad as we assume?”), doubts about ingroup favoritism (“Are we and our ingroup really as good as we assume?”), and doubts about the intergroup inequality assumption (“Are we really as different from the outgroup as we assume?”). Interventions to explicitly reduce homonegativity that have been implemented and tested to date can also, to a lesser extent, be categorized into cognitive, relational, and institutional level interventions proposed by Hartman et al. (2022; see also Jewell et al., 2011). A comparable systematic review relevant to prejudice reduction research (Paluck & Green, 2009) indicates that laboratory experimental approaches based on social identity theoretical assumptions are as relevant as cognitive training (p. 358). In the present study, we developed a metacognitive training (MCT) based on social identity theoretical assumptions. In other words: We induced social identification-based metacognitive doubt about ingroup favoritism and outgroup derogation to evoke an “aha”-experience possibly leading to intellectual humility which should be observable in reduced homonegativity and extended outgroup tolerance among heterosexual Republicans.

### The Present Study: Metacognitive Training as a Tool for Reducing Modern Homonegativity in Republican Leaners

In the present study, we aimed to investigate an intervention strategy that stems from clinical work with individuals with psychosis/schizophrenia: metacognitive training. MCT attempts to evoke an “aha” moment in MCT participants in a playful way and to open up new perspectives (i.e., doubts about former, especially wrong held views and beliefs). As central mechanisms of action, MCT raises awareness for cognitive biases and reduces overconfidence through “aha” experiences (Köther et al., 2017; Moritz et al., 2019, 2023). In MCT, seemingly easy questions are posed with appropriately framed response options that elicit stereotype-conform, wrong response tendencies in the respondent. Following this, feedback is given, which is usually intended to elicit and open up irritation and an “aha” moment (that is, metacognitive doubt) in MCT participants. According to a recent review encompassing 43 studies, medium to large effects could be achieved in patients with schizophrenia on primary outcomes (Penney et al., 2022). Importantly, however, this intervention was successfully applied in non-clinical, social groups and produced intellectual humility as evidenced by reduced prejudice and intergroup animosity: In Christians and Muslims, mutual prejudice was successfully reduced (Moritz et al., 2018, 2021), and in U.S.-American Liberals, prejudice against conservatives was reduced (Reininger et al., 2020a).

More specifically, we raised doubts about ingroup favoritism (i.e., identification-based metacognitive doubt; Reininger et al., 2020a) in U.S. liberals through MCT items which in turn might have induced intellectual humility. One example item—that was incorrectly assessed by around 50% of MCT participants—was the question about air strikes (largely carried out by drones) in relation to the covert action against terrorism. The answer “There were 10 times more air strikes during Obama’s Presidency than during Bush’s Presidency” was incorrectly assessed by 49.5% of the participating liberals (to the advantage of the Obama administration). On the other hand, and in particular, we have raised doubts about outgroup derogation. For example, we might have induced intellectual humility toward the conservative outgroup with the MCT item “How many Republicans say there should be a way for undocumented immigrants to stay in the country, if certain requirements are met?” Only 12.9% answered correctly (“*56%*”)—in other words, over 87% made an incorrect assumption about the conservative outgroup. As a result of the intervention (and compared to an active education and a no-treatment control condition), participants in the MCT condition improved in terms of affective polarization (i.e., less negative feelings toward conservatives, perception of threat) and cognitive polarization (i.e., more positive evaluation and stereotyping of conservatives).

The MCT condition usually consists of MCT items, each comprising one question with four answer options. The majority of the MCT items used should include questions on topics that address a false stereotype. For example, in the latter example of the question about Republicans’ attitudes toward legal pathways for undocumented immigrants to stay in the country, we chose the response options in a way that the correct answer (56%) was the highest answer option. The other answer options (7%, 14%, and 37%) aimed to prime liberal participants to underestimate the level of Republicans’ kindness toward refugees. In contrast, at the beginning of the MCT condition, there are always so-called primer questions, in which we do not use contrastereotypical content, but content that is regularly answered correctly by the participants. All MCT items (question with answer options) are provided with feedback at the end (“The answer you have chosen is correct vs. incorrect.”) and then an information text about the correct answer. Understandably, a well-suited active control condition is the presentation of the information alone, without any prior metacognitive question–answer game (i.e., the education condition).

Thus, with respect to the group of heterosexual Republicans, in the developed MCT we selected questions (including answers and informational texts) that should evoke doubts about the ingroup favoritism of heterosexual Republicans and doubts about outgroup derogation of LGBTIQ+ persons which also might pave the way for participants’ intellectual humility.

## Method

### Design

We applied a randomized controlled between-subjects design with three conditions by randomly assigning participants either to the MCT condition (*n* = 166), the education condition (*n* = 164), or to the no-treatment condition (*n* = 160). Our study was preregistered by the German Clinical Trial Register (DRKS00029115).

### Power Analysis

We based our power analysis on the assumption that the three conditions (metacognitive training vs. education vs. no treatment) would exert a small to medium effect (i.e., *f* = 0.175, *d* = 0.35) on the dependent variables (in line with the results observed in Reininger et al., 2020a). We applied this effect size to an a priori power analysis for three groups within an ANOVA. The power analysis indicated that approximately 510 participants would be needed to achieve 95% power (1 - *β*) at a .05 alpha level (*α* = .05).

### Participants

To account for potential study dropouts, we recruited a convenience sample of 629 adult U.S.-American citizens online via *CloudResearch,* who were supposed to be Republicans or Republican Leaners (Litman et al., 2017). *CloudResearch* draws on previously screened participants from *Amazon’s Mechanical Turk* (Buhrmester et al., 2011). A total of 34 participants were excluded because they answered less than 80% questions of the total study questions. Forty-four other participants were excluded because they answered “Democratic Party” to the question “Do you think of yourself as closer to the Republican or Democratic party?” Twenty-one other participants were excluded because they indicated a score between 1 and 5 (that is, within the liberal range) on a 10-point Likert scale ranging from 1 (*extremely liberal*) to 10 (*extremely conservative*) in response to the question “Where on the following scale of political orientation would you place yourself?” Sixteen other participants were excluded because they indicated a score less than 4 with regard to the item “I was focused while filling out this survey.” on a Likert scale ranging from 1 (*No agreement at all*) to 5 (*Extremely agree*). Finally, we excluded 24 people who did not indicate heterosexual when asked about sexual orientation (with one exception: One person indicated something else not listed as sexual orientation and wrote “straight” in the free text box, which we interpreted as heterosexual orientation). Thus, our final sample included 490 participants who identified with the Republican Party (323 female and 167 male). The mean age was 47 years (*SD* = 14.32). Most identified themselves as Caucasian (86.7%), with 2.7% as African-American, 3.1% as Asian-American, 4.3% as Hispanic American, 2.7% as multiracial American, and 0.6% as other. Participants did not differ with regard to the above-mentioned and further sociodemographic variables across conditions (Table 1).

**Table 1:** Demographic data of participants by condition

Variable	Metacognitive training (*n* = 166)		Education (*n* = 164)		No treatment (*n* = 160)		Difference between conditions
	*n*	*%*	*n*	*%*	*n*	*%*	
Gender							
Male	55	33.1	59	36.0	53	33.1	χ^2^(2) = 0.39, *p* = .821
Female	111	66.9	105	64.0	107	66.9	
Sexual orientation							
Heterosexual	166	100	164^a^	100	160	100	
Relationship status^b^							
Single	42	25.3	28	17.1	33	20.6	χ^2^(12) = 11.12, *p* = .518
Life partner	5	3.0	4	2.4	7	4.4	
Romantic relationship	4	2.4	12	7.3	10	6.3	
Engaged	3	1.8	3	1.8	3	1.9	
Married	94	56.6	99	60.4	83	51.9	
Divorced/separated	11	6.6	13	7.9	18	11.3	
Other	6	3.6	5	3.0	6	3.8	
Ethnic background							
Asian/Asian-American	9	5.4	5	3.0	1	0.6	χ^2^(10) = 10.61, *p* = .389
Black/African-American	4	2.4	6	3.7	3	1.9	
Latino/Hispanic	8	4.8	7	4.3	6	3.8	
White/European American	142	85.5	141	86.0	142	88.8	
Other	1	0.6	1	0.6	1	0.6	
More than one	2	1.2	4	2.4	7	4.4	
Education							
No diploma	2	1.2	2	1.2	1	0.6	χ^2^(12) = 12.7, *p* = .391
High-school diploma	29	17.5	19	11.6	19	11.9	
Some college	43	25.9	31	18.9	40	25.0	
Associate degree	24	14.5	34	20.7	25	15.6	
Bachelor’s degree	52	31.3	53	32.3	55	34.4	
Master’s degree	15	9.0	19	11.6	18	11.3	
Professional degree or doctorate degree	1	0.6	6	3.7	2	1.3	
Political orientation^c^		*M*(SD)		*M*(SD)		*M*(SD)	
		8.27 (1.29)		8.18 (1.35)		8.41 (1.29)	χ^2^(8) = 5.87, *p* = .662
Age		45.84 (13.63)		46.36 (13.76)		49.53 (15.36)	χ^2^(122) = 123.74, *p* = .439

^a^One participant provided the term *straight* after selecting the option *Other*. This participant was noted under the category *Heterosexual*

^b^In the metacognitive training condition, one response was missing (0.6%)

^c^For this, a 10-point Likert-type scale was employed (1 = *extremely liberal* to 10 = *extremely conservative*)

### Procedure

#### Informed Consent and Initial Questions

Participants completed the study on Qualtrics, a web-based tool for creating surveys. Before Mechanical Turk users were randomly assigned to one of the three conditions, they provided informed consent and answered to measures of political orientation (e.g., “Do you think of yourself as closer to the Republican or Democratic party?”). After the intervention (or the no treatment), they were directly led to the dependent measures.

#### Metacognitive Training Condition

In the metacognitive training condition, we asked two questions which included—from the perspective of heterosexual Republican participants—correct stereotypical information (filler questions) such as “Why do Christians believe in the importance of marriage?” with four choices where one is obviously the correct one (“Marriage is one of the seven sacraments and aims for the good of each other and the procreation of children”); for all items in the MCT condition, see Table 2.

**Table 2:** Questions, responses, and endorsement in the metacognitive training condition

Question	Response option	Endorsement present study (%)
1. Why do Christians believe in the importance of marriage?	- Marriage is one of the seven sacraments and aims for the good of each other and the procreation of children [correct]	106 (63.9%)
	- Partners feel more connected	2 (1.2%)
	- It is a sign of love and commitment	58 (34.9%)
	- It offers financial advantages	0
2. What does GOP stand for?	- Government of the People	48 (28.9%)
	- Grand Old Party [correct]	111 (66.9%)
	- God Ordained Party	1 (0.6%)
	- Governmental Party	6 (3.6%)
3. In how many animal species worldwide could same-sex behavior be observed?	- In none animal species	27 (16.3%)
	- In less than 5 animal species	38 (22.9%)
	- In less than 10 animal species	32 (19.3%)
	- In over 450 animal species [correct]	69 (41.6%)
4. Cornell University in New York identified 79 scientific studies focusing on well-being of children with gay or lesbian parents. Which of the answer options do you think is most likely?	- No study could accurately determine if children with same-sex parents are better/worse off	64 (38.6%)
	- Only four of 79 studies show that children tend to fare worse with same-sex parents [correct]	41 (24.7%)
	- 75 studies show that children with same-sex parents fare worse due to discrimination	17 (10.2%)
	- Half of the studies are indecisive	44 (26.5%)
5. How many Republicans and Republican leaners favor same-sex marriage in 2019?	- 3%	36 (21.7%)
	- 12%	62 (37.3%)
	- 36%	37 (22.3%)
	- 44% [correct]	31 (18.7%)
6. How many U.S. Catholics say society should be accepting of homosexuality?	- 8%	72 (43.4%)
	- 15%	49 (29.5%)
	- 37%	29 (17.5%)
	- 76% [correct]	16 (9.6%)
7. How many Republicans claimed in 2021 that a person can be a man or a woman even if that is different from the sex they were assigned at birth?	- 0,6%	64 (38.6%)
	- 3%	57 (34.3%)
	- 8%	29 (17.5%)
	- 17% [correct]	16 (9.6%)
8. How many transgender people are in the U.S. military?	- no documented persons	63 (38.0%)
	- 600	67 (40.4%)
	- 8,900	28 (16.9%)
	- 15,500 [correct]	8 (4.8%)
9. How many of the people supporting Democrats are gay, lesbian, or bisexual?	- 6.3% [correct]	21 (12.7%)
	- 18.7%	24 (14.5%)
	- 22.3%	28 (16.9%)
	- 37.2%	93 (56.0%)
10. How many Democrats and Democrat leaners say society has gone too far in accepting transgender people?	- 1%	72 (43.4%)
	- 3%	50 (30.1%)
	- 7%	28 (16.9%)
	- 12% [correct]	16 (9.6%)
11. How many adults in the U.S. identify as LGB (i.e., lesbian, gay, or bisexual)?	- 3.5% [correct]	48 (28.9%)
	- 7.8%	36 (21.7%)
	- 12.3%	46 (27.7%)
	- 18.2%	36 (21.7%)
12. How many adults in the U.S. identify as transgender?	- 0.001%	30 (18.1%)
	- 0.3% [correct]	54 (32.5%)
	- 1.2%	45 (27.1%)
	- 7.2%	37 (22.3%)
13. How did adolescents with lesbian mothers rate their well-being on a 10-point-maximum scale (ranging from 1 = *poorest possible* to 10 = *highest possible*)?	- 3.38	22 (13.3%)
	- 5.75	68 (41.0%)
	- 7.12	48 (28.9%)
	- 8.14 [correct]	28 (16.9%)
14. How many LGBT voters helped reelect George W. Bush in 2004?	- no LGBT voters	24 (14.5%)
	- 1% of all LGBT voters	81 (48.8%)
	- 13% of all LGBT voters	47 (28.3%)
	- 25% of all LGBT voters [correct]	14 (8.4%)
15. Some LGB people undergo conversion therapy in order to change their sexual orientation. To what extent is LGB persons’ risk of contemplating or attempting suicide increased when they undergo conversion therapy compared to LGB peers who have not experienced conversion therapy?	- Significantly lower risk, as conversion therapy reduces suicides	27 (16.3%)
	- No higher risk	37 (22.3%)
	- Slightly increased	58 (34.9%)
	- Almost twice as likely [correct]	44 (26.5%)
16. How many times are gay people more likely to report having attempted suicide compared with peers from families that reported no or low levels of family rejection?	- 1.5 times	38 (22.9%)
	- 3.2 times	46 (27.7%)
	- 5.7 times	53 (31.9%)
	- 8.4 times [correct]	29 (17.5%)

*Note* The percentages are rounded values, so the total may be more or less than 100% due to the rounded percentages

Subsequently, we posed 14 MCTs. The purpose of these MCTs was to raise doubts regarding the three theoretical frames of reference already mentioned above: ingroup favoritism, outgroup derogation, or intergroup inequality assumption. For example, one MCT targeting outgroup derogation of LGBTIQ+ persons asked: “How many transgender people are in the U.S. military?” with response—options (1) “no documented persons,” (2) “600,” (3) “8,900,” or (4) “15,500.” Indeed, 95.3% of our participants in the MCT condition did not select the correct answer option (4) (i.e., 15,500 persons); this corresponds to the (counterfactual, inaccurate) belief that transgender persons were not serving in the military. After the 14 MCT questions with answer options, participants in the MCT condition were presented with the original question, their chosen answer and the following feedback for every MCT item. For example: “Your answer was wrong! 15,500 transgender adults are serving in the U.S. military, Reserves, or National Guard. Source: https://williamsinstitute.law.ucla.edu/quick-facts/lgbt-faqs/.”

All MCT questions, answer options, and answer distributions of the participants in the MCT condition are given in Table 2.

#### Education Control Condition

In parallel to the above-mentioned doubt induction of transgender adults serving in the military, in our active control condition (education control condition), only the informational texts were presented for at least 10 s (i.e., “Please read the following information: 15,500 transgender adults are serving in the U.S. military, Reserves, or National Guard”). Source: https://williamsinstitute.law.ucla.edu/quick-facts/lgbt-faqs/”).

#### No-Treatment Control Condition

In the no-treatment condition, no intervention was provided.

### Measures

As dependent variables, we assessed homonegativity and the perception of being threatened by LGBTIQ+ persons.^1^

#### Homonegativity

We assessed homonegativity with the Modern Homonegativity Scale (Morrison & Morrison, 2003). The Modern Homonegativity Scale comprises 12 items and showed excellent internal consistency (*α* = 0.93). The term “gay men” was replaced by “LGBTIQ+” in all statements. Example items are “LGBTIQ+ use their sexual orientation so that they can obtain special privileges,” “LGBTIQ+ should stop complaining about the way they are treated in society and simply get on with their lives,” and “LGBTIQ+ should stop shoving their lifestyle down other people’s throats.” The items could be answered with a slider from 0 (*strongly disagree*) to 100 (*strongly agree*).

#### Perception of Being Threatened by LGBTIQ+-Persons

We assessed the Republican leaner participants’ perception of being threatened by LGBTIQ+-persons with two items, *r*(488) = .77, *p* ≤ .001: “Are the following people a threat to U.S. society?—LGBTIQ+-persons in the U.S.” (Reininger et al., 2020a) and “Would you say the LGBTIQ+ community with its views and practices is so misguided that they threaten the nation’s well-being?” (Pew Research Center, 2014) both on a Likert scale ranging from 0 (*not at all*) to 4 (*absolutely*).

#### Tolerance Toward Other Outgroups

In order to obtain an indicator of tolerance (i.e., disapproval being tamed by respect; Simon, 2020, 2023; Simon et al., 2019; Zitzmann et al., 2022) in our Republican leaner participants toward relevant outgroups, we asked (in line with Simon et al., 2019):

“Should the following people be allowed to live the way they want to live?” With the target groups: “LGBTIQ+ persons in the U.S.,” “Feminists in the U.S.,” “Liberals in the U.S.,” and “Climate Activists in the U.S..” For each group, participants could rate their level of tolerance on a 5-point Likert scale ranging from −2 (*not at all*) to + 2 (*absolutely*). The scale of these ratings showed an excellent reliability of *α* = 0.92.

### Analytic Strategy

We submitted the dependent measures to an analysis of variance (ANOVA) with condition (no treatment vs. education vs. MCT) as fixed between-subject factor. As post hoc analysis, we calculated the least significant difference (LSD).

## Results

In the first two MCT items, we asked non-irritating, easy-to-answer questions with stereotypically conforming questions resulting in more than 60% of our participants choosing the correct response options. In the next 14 MCTs, we developed to induce doubt in our participants, we observed that this pattern reversed: The following MCTs had sufficiently high difficulty that at least 58% of MCT condition participants gave an incorrect answer. The exact distribution of participants between the correct and incorrect response options is shown in Table 2 and all statistical results in Table 3.

**Table 3:** Comparison of the dependent measures between the conditions (no treatment vs. education vs. MCT)

Scale	No treatment (*n* = 160)		Education (*n* = 164)		Metacognitive training (*n* = 166)				No treatment vs. metacognitive training		Education vs. metacognitive training		No treatment vs. education	
	*M*	*SD*	*M*	*SD*	*M*	*SD*	*F*(2, 487)	*p*	*p*^a^	*d*	*p*^a^	*d*	*p*^a^	*d*
Homonegativity (*Modern Homonegativity Scale*)	73.38	20.75	73.45	21.23	67.22	22.74	4.52	.011	.010	0.28	.009	0.28	.978	0.00
Perception of threat	2.08	1.34	2.07	1.29	1.69	1.23	4.90	.008	.007	0.30	.007	0.30	.955	0.01
Tolerance with regard to other groups	0.88	1.20	0.86	1.19	1.13	0.98	3.06	.048	.044	0.23	.026	0.26	.835	0.02

The *Modern Homonegativity Scale* is captured by the item “Use the slider to indicate whether you agree or disagree with the following statements (0 = *strongly disagree* to 100 = *strongly agree*).” The scale *Perception of Threat* is captured by the items “Are the following people a threat to U.S. society?—LGBTIQ+ persons in the U.S. (0 = *not at all* to 4 = *absolutely*)” and “Would you say the LGBTIQ+ community with its views and practices is so misguided that it threatens the nation’s well-being? (0 = *not at all* to 4 = *absolutely*).” The scale *Tolerance With Regard to Other Groups* is captured by the items “Should the following people be able to live the way they want to live?—LGBTIQ+ persons in the U.S (−2 = *not at all* to + 2 = *absolutely*),” “Should the following people be able to live the way they want to live?—Feminists in the U.S (−2 = *not at all* to + 2 = *absolutely*),” “Should the following people be able to live the way they want to live?—Liberals in the U.S. (−2 = *not at all* to + 2 = *absolutely*),” and “Should the following people be able to live the way they want to live?—Climate activists in the U.S. (−2 = *not at all* to + 2 = *absolutely*)”

^a^Applying* t*-tests

### Homonegativity

**Fig. 1:**
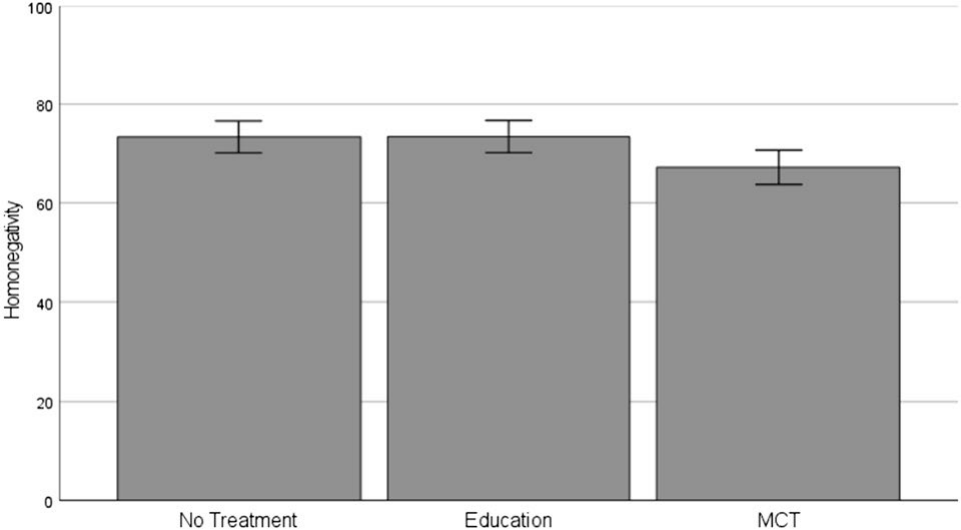
Comparison of homonegativity between the conditions (no treatment vs. education vs. MCT): Means and 95% confidence intervals as error bars

We observed a significant difference in homonegativity between the three conditions, *F*(2, 487) = 4.52, *p* = .011, *η*^2^ = 0.02. Participants in the MCT condition, *M* = 67.22, *SD* = 22.74, showed a lower amount of homonegativity, than those in the education condition, *M* = 73.45, *SD* = 21.23, *t*(487) = 2.62, *p* = .009, *d* = 0.28, 95% CI [0.07; 0.50], and those in the no-treatment condition, *M* = 73.38, *SD* = 20.75, *t*(487) = 2.58, *p* = .010, *d* = 0.28, 95% CI [0.06; 0.50] (Fig. 1). Participants in the education condition did not differ from those in the no-treatment condition, *p* = .978.

### Perception of Being Threatened by LGBTIQ+ Persons

**Fig. 2:**
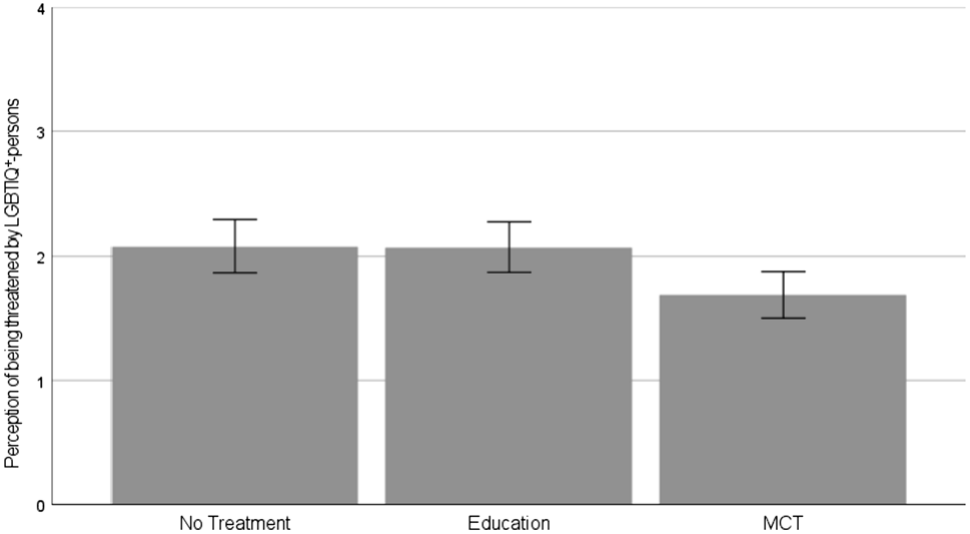
Comparison of the perception of being threatened by LGBTIQ+ people between the conditions (no treatment vs. education vs. MCT): Means and 95% confidence intervals as error bars

We observed a difference between the three conditions, *F*(2, 487) = 4.90, *p* = .008, *η*^2^ = 0.02. Participants in the MCT condition, *M* = 1.69, *SD* = 1.23, showed a lower perception of being threatened by LGBTIQ+ persons than those in the education condition, *M* = 2.07, *SD* = 1.29, *t*(487) = 2.69, *p* = .007, *d* = 0.30, 95% CI [0.09; 0.52], and those in the no-treatment condition, *M* = 2.08, *SD* = 1.34, *t*(487) = 2.73, *p* = .007, *d* = 0.30, 95% CI [0.08; 0.52] (Fig. 2). Participants in the education condition did not differ from those in the no-treatment condition, *p* = .955.

### Tolerance Toward Other Outgroups

**Fig. 3:**
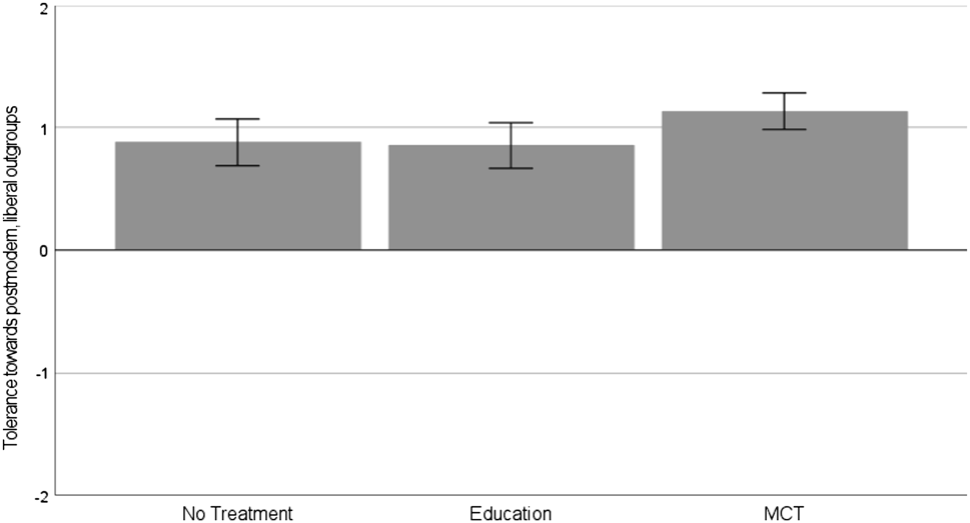
Comparison of tolerance toward other outgroups between the conditions (no treatment vs. education vs. MCT): Means and 95% confidence intervals as error bars

We observed a difference between the three conditions, *F*(2, 487) = 3.06, *p* = .048, *η*^2^ = 0.01. Participants in the MCT condition, *M* = 1.13, *SD* = 0.98, showed more tolerance toward other outgroups than those in the education condition, *M* = 0.86, *SD* = 1.19, *t*(487) = 2.24, *p* = .026, *d* = 0.26, 95% CI [0.04; 0.47], and those in the no-treatment condition, *M* = 0.88, *SD* = 1.20, *t*(487) = 2.02, *p* = .044, *d* = 0.23, 95% CI [0.01; 0.45] (Fig. 3). Participants in the education condition did not differ from those in the no-treatment condition, *p* = .835.

## Discussion

In the present study, we observed that our developed MCTs exerted small (and at times small to medium) but significant effects on homonegativity, threat perceptions of LGBTIQ+ individuals as well as in a broader sense extended tolerance toward other outgroups compared to two control conditions, one of them being an active control, in U.S. Republican Leaners. For this, we presented two short MCTs eliciting stereotype-conforming correct responses and fourteen short MCTs eliciting stereotype-conforming incorrect responses followed by informative correction.

The small effects are consistent with those we have observed in previous studies in intergroup settings, such as in the studies with Christians and Muslims in which mutual prejudice was successfully reduced (Moritz et al., 2018, 2021), and in the study with U.S. liberals, in which prejudice against conservatives was reduced (Reininger et al., 2020a). The study tentatively suggests that the specific type of metacognitive training may be a promising way to depolarize polarization processes compared to simple information presentation (education control condition) or no treatment.

Nevertheless, there are also limitations of this study: The final sample deviated—for reasonable exclusions—from the originally planned number by 20 participants and for future studies we should definitely recruit larger samples (with the negligible risk of too much power). We were also not able to provide any information on the impact of our small to partly medium effects. The question of whether these have a longer-term effect or only bring about a short-term change in attitude should be described by future research.

In its preliminary nature, the study should be interpreted as a pilot study that nonetheless goes beyond the scope of previous studies in religious or liberal participant populations: To our knowledge, it is the first to apply metacognitive training in a conservative group (in contrast to previous studies in liberal or religious contexts). However, choosing republican leaners as research participants and LGBTIQ+ persons as main target of the MCT-intervention while not controlling for religious orientation is a main point of criticism. Interestingly, by raising metacognitive identification-based doubt in our MCT items, we might have induced intellectual humility (which unfortunately was not assessed with a manipulation check item). Intellectual humility, in turn, might explain the observed “spillover effects” to other (disapproved) outgroups (with regard to the extended-tolerance items). This is an interesting and comprehensible, but preliminary finding: Inducing intellectual humility via identification-based metacognitive doubt reduces homonegativity and threat perceptions of LGBTIQ+ individuals and is accompanied by increased extended tolerance toward other disapproved outgroups. In other words: Although Republican leaners do not homogeneously disapprove of LGBTIQ+ people, our metacognitive identification-based doubt induction might have lead to intellectual humility, which on the one hand reduced homonegativity and threat experiences of LGBTIQ+ people at group level, but on the other hand might have promoted extended tolerance toward distinct other outgroups such as feminists or liberals in the USA.

The mechanisms at work here are not fully understood: We have developed different metacognitive training items ourselves and can assign them to the categories of “doubt about ingroup favoritism” and “doubt about outgroup derogation”—though we do not yet know which of these categories is likely to exert the best effects. Nevertheless, this pilot study adds some relevant points to the existing state of research. MCT as a method theoretically grounded in its emphasis on thinking about thinking has been applied in a third relevant social group (religious groups, a liberal group, and now a conservative group). As the content of this training, we have used counter-stereotypical information that might have established intellectual humility through doubts about ingroup favoritism and outgroup derogation (i.e., identification-based metacognitive doubt). In this respect, the effects of these types of identification-based metacognitive doubt, which might have fostered intellectual humility, should not be underestimated: They have reduced polarization compared to an active and a no-treatment control condition, but above all they have also reduced polarization in the sense of intellectual humility toward completely different social groups that were not affected by the content of the metacognitive training. This stance of intellectual humility poses important questions for future experimental MCT studies: Does any metacognitive doubt promote intellectual humility and, if so, how far (for example, up to which rejected outgroups and how sustainable) is it still effective? Furthermore, the question arises as to how long such an intervention is effective at all. In the literature, MCT has shown that raising awareness of the cognitive biases involved in the pathogenesis of psychosis and reducing overconfidence through “aha” experiences are central mechanisms of action in the reduction of symptoms. The present pilot study with our adaptation of MCT in the non-clinical, social, and societal area contributes to one realization in particular: MCT might be able to promote intellectual humility and reduce overconfidence, which has the potential for radicalized polarization in intergroup contexts (e.g., Reininger, 2018; Reininger et al., 2024). Social group members obviously become more cautious, more tamed, more humble, less prejudiced, and less polarized in their assessment of outgroup members through MCT. The specific technique of the question–answer game, with its “correct,” “aha”-moments evoking, intellectual humility-promoting content of doubting ingroup favoritism and outgroup derogation, is probably a useful way to counter increasing polarization. This adds a crucial aspect to the existing interventions mentioned by Paluck and Green (2009) as well as Hartman et al. (2022): MCT evoked metacognitive doubt seems to create intellectual humility being able to exert a taming effect on intergroup hostility (for example, in the expression of cognitive or affective polarization) and possibly fostering dynamics of respect (Reininger et al., 2020b).

To conclude, such metacognitive training seems to be applicable in institutions to reduce disapproval (in the sense of homonegativity) and threat perception and to promote tolerance (also in the broader sense). If successful, this may have indirect effects on stigmatization and thus on health of LGBTIQ+ people. In a broader understanding, however, such interventions could also be particularly helpful in bringing the self-referential echo chambers (Brady et al., 2017) of our polarized postmodern society into contact, thus promoting the prerequisite of democratic societies, namely communication between groups.

## Data Availability

On reasonable request, data are available from the corresponding author.

## Footnotes

1 The questionnaire contained additional measures relevant to other research questions of the larger project. However, all data analyses reported in the article are novel and the findings have not been published elsewhere.
